# Morphine Binds Creatine Kinase B and Inhibits Its Activity

**DOI:** 10.3389/fncel.2018.00464

**Published:** 2018-12-03

**Authors:** Ivan Weinsanto, Jinane Mouheiche, Alexis Laux-Biehlmann, François Delalande, Arnaud Marquette, Virginie Chavant, Florian Gabel, Sarah Cianferani, Alexandre Charlet, Marie-Odile Parat, Yannick Goumon

**Affiliations:** ^1^Institut des Neurosciences Cellulaires et Intégratives, CNRS UPR3212 and Université de Strasbourg, Strasbourg, France; ^2^Laboratoire de Spectrométrie de Masse BioOrganique, IPHC-DSA, CNRS UMR7178 and Université de Strasbourg, Strasbourg, France; ^3^CNRS UMR7177 and Université de Strasbourg, Strasbourg, France; ^4^Mass Spectrometry Facilities of the CNRS UPR3212, Strasbourg, France; ^5^School of Pharmacy, University of Queensland, PACE, Woolloongabba, QLD, Australia

**Keywords:** morphine, complex, ligand-binding protein, creatine kinase, high affinity

## Abstract

Morphine is an analgesic alkaloid used to relieve severe pain, and irreversible binding of morphine to specific unknown proteins has been previously observed. In the brain, changes in the expression of energy metabolism enzymes contribute to behavioral abnormalities during chronic morphine treatment. Creatine kinase B (CK-B) is a key enzyme involved in brain energy metabolism. CK-B also corresponds to the imidazoline-binding protein I_2_ which binds dopamine (a precursor of morphine biosynthesis) irreversibly. Using biochemical approaches, we show that recombinant mouse CK-B possesses a μM affinity for morphine and binds to morphine *in vitro*. The complex formed by CK-B and morphine is resistant to detergents, reducing agents, heat treatment and SDS-polyacrylamide gel electrophoresis (SDS-PAGE). CK-B-derived peptides CK-B_1–75_ and CK-B_184–258_ were identified as two specific morphine binding-peptides. *In vitro*, morphine (1–100 μM) significantly reduces recombinant CK-B enzymatic activity. Accordingly, *in vivo* morphine administration (7.5 mg/kg, i.p.) to mice significantly decreased brain extract CK-B activity compared to saline-treated animals. Together, these results show that morphine strongly binds CK-B and inhibits its activity *in vitro* and *in vivo*.

## Background

Morphine, an alkaloid from *Papaver somniferum*, is used to relieve pain in multiple clinical settings. In addition to its analgesic properties, morphine decreases intestinal motility, suppresses cough and has vasodilatory effects (Andersen et al., 2003). It influences many other physiological processes and notably decreases ATP availability in specific brain structures (Nasello et al., 1973). In addition, endogenous morphine has been characterized in numerous mammalian cells and tissues, and its structure is identical to that of morphine isolated from the poppy (for review Laux-Biehlmann et al., 2013).

Only a few unspecific morphine-binding proteins with low affinity have been identified so far and include serum albumin (Judis, 1977; Leow et al., 1993). In the past, our laboratory has demonstrated that the phosphatidylethanolamine-binding protein (PEBP; Goumon et al., 2004) binds to morphine-6-glucuronide (M6G) and morphine-3-glucuronide (M3G) with an affinity equal to that of its reference ligand, phosphatidylethanolamine, but has no affinity for morphine (Atmanene et al., 2009). In addition to these low affinity binding-proteins, covalent binding of morphine to proteins has been proposed to result in irreversible binding to insoluble tissue components after administration (Misra et al., 1971; Mullis et al., 1979; Nagamatsu et al., 1983).

The implication of the imidazoline system, and particularly the I_2_-binding site (I2B; Li, 2017), in the modulation of morphine-induced analgesia and morphine analgesic tolerance has been documented (Gentili et al., 2006; Caprioli et al., 2015). Interestingly, creatine kinase B (CK-B) represents the main I2B site for imidazoline and its derivatives (clonidine, guanfacine; Kimura et al., 2009; Li, 2017). Molecular modeling using the crystal structure of chicken CK-B and the irreversible ligand BU990006-binding site has indicated the importance of Thr_71_, Val_72_, Val_75_, Leu_201_, Leu_202_, Cys_283_ and Ser_285_ in this interaction (Kimura et al., 2009). Dopamine, a morphine precursor in plants and mammals (Laux-Biehlmann et al., 2013), has been shown to covalently bind to CK-B (Van Laar et al., 2009). CK-B also forms highly stable complexes with ankyrin repeat and SOCS box protein 9 (ASB9; Balasubramaniam et al., 2015).

CK-B is a 42 kDa protein expressed in neurons, oligodendrocytes and astrocytes of the central nervous system (CNS; Manos and Bryan, 1993). Different CK isoenzymes exist in the cytoplasm as dimers or multimers in specific cells: CK-BB in the brain, CK-MM in muscles and CK-MB in the heart (Wyss and Kaddurah-Daouk, 2000). CK catalyzes the reversible phosphorylation of creatine by ATP to produce phosphocreatine and ADP. Mitochondrial isoforms of CK form phosphocreatine from ATP, whereas cytoplasmic CK isoenzymes form ATP from phosphocreatine.

In the present study, we have investigated whether CK-B might represent a morphine-binding protein. Recombinant mouse CK-B (rCK-B) and mouse CK-B-derived synthetic peptides, together with biochemistry approaches, were used to characterize the affinity of potential morphine-binding sites. To assess the functional significance of CK-B/morphine interactions, the impact of morphine on rCK-B activity was studied *in vitro* and *in vivo*.

## Materials and Methods

### Experimental Design

Our animal study is reported in accordance with the ARRIVE Guidelines for reporting experiments involving animals (McGrath et al., 2010). Experiments were carried out in a randomized and blind manner, and statistical analyses were done prior to revealing treatment groups. At least three technical replicates were used for *in vitro* experiments. Mice were assigned an identity number and assigned to groups randomly so that the experimenter was blind to treatment.

### Drugs

Morphine base was purchased from Euromedex (Souffelweyersheim, France). A stock solution of 35 mM morphine was prepared by dissolving morphine in H_2_O after adding an equimolar concentration of HCl. Codeine monohydrate, morphine-3-β-D-glucuronide and morphine-6-β-D-glucuronide dihydrate powders were purchased from Sigma-Aldrich (Lyon, France) and dissolved in H_2_O to prepare stock solutions. Working concentrations for *in vitro* and *in vivo* experiments were prepared from the stock on the day of experiment. When necessary, the pH of the working solutions was adjusted to 7.4 with NaOH before use.

### Animals

All procedures were performed in accordance with European directives (86/609/EEC) and were approved by the regional ethics committee and the French Ministry of Agriculture (license No. 00456.02 to YG). Experiments were performed with C57BL/6 mice (45 day-old adult male, 24 ± 3 g; Charles River, L’Arbresle, France). Animals were given food and water *ad libitum*, and maintained in a 12 h light–dark cycle at a room temperature of 22°C ± 2°C. Cage bedding was from Anibed (Pontvallain, France; reference AB3) and food from SAFE (Augy, France; reference A04). Mice were kept group-housed at five per cage (Type II cage, 370 cm^2^, height 14 cm).

### Drug Injections

Mice were weighed and then i.p. injected (light phase at 10 AM) with a single dose of 7.5 mg/kg morphine or an equivalent volume of saline (NaCl 0.9% in H_2_O). The volume of injection was 240 ± 30 μL for mice weighing 24 ± 3 g. After 90 min, mice were anesthetized with a solution of ketamine and xylazine (17 mg/mL ketamine and 2.5 mg/mL xylazine, 4 mL/kg i.p.; Centravet, Taden, France). After 5 min, adequate anesthesia was ensured by pinching the hind paws and observing no reflex response. Animals were then euthanized by decapitation. Brains were then collected and frozen at −80°C.

### Preparation of Brain Extracts From Morphine and Saline-Treated Mice

Brains from morphine- and saline-treated mice were homogenized with an Ultra Turrax instrument (Ika, Staufen, Germany) in 1 ml of H_2_O containing protease inhibitors (cOmplete Mini, EDTA-free, Roche, Basel, Switzerland). The homogenates were then sonicated (two times 10 s, 90 W) with a Vibra Cell apparatus (Sonics, Newtown, CT, USA) and centrifuged (14,000× *g*, 30 min) at 4°C. Supernatants were recovered and protein concentration was determined using the Bradford method (Protein Assay, Bio-Rad, Marnes-la-Coquette, France). Samples were then frozen at −80°C until further use.

### Production of the Mouse rCK-B

The N-terminal His-tagged cDNA coding region for the mouse Ckb (residues 1-318, accession number NM_021273; ref. MR205953, Origene, Herford, Germany**)** was cloned into the vector pET15b (CIGEx, CEA, Fontenay aux Roses, France). The N-terminal hexahistidine-tagged fusion protein was expressed in *Escherichia coli* BL21 (DE3). Cells were grown in LB medium (Euromedex) at 37°C until an A_600_ of 0.4 and subsequently induced for 3 h at 37°C with 0.1 mM isopropyl 1-thio-D-galactopyranoside (Euromedex). Harvested bacterial pellets were resuspended in buffer containing isopropyl thio-β-d-galactoside (IPTG; Euromedex). The cell pellets were resuspended in binding buffer (10 mM Tris-HCl, 300 mM NaCl, 10% glycerol, 2 mM CHAPS and 10 mM imidazole, pH 8.0; Euromedex), lysed by sonication on ice (8 × 30 s, 90 W) and then clarified by centrifugation (20,000× *g*, 50 min, 4°C). The cleared supernatant was loaded on a nickel-Hitrap column (GE Healthcare; Aulnay Sous Bois, France). The protein was eluted using 50 and 250 mM imidazole (10 mM Tris-HCl, 300 mM NaCl, pH 8.0; Euromedex). The protein was then dialyzed 12 h at 4°C against a buffer containing 10 mM Tris, and 150 mM NaCl (pH 8). The purity and homogeneity of the protein were assessed by SDS-polyacrylamide gel electrophoresis (SDS-PAGE; see below).

### Enzyme-Linked ImmunoSorbent Assay (ELISA)

Enzyme-linked immunosorbent assays (ELISAs) were performed to determine the affinity and specificity of the binding of mouse rCK-B and its derived peptides to opiate alkaloids. 96-well plates (NUNC, Roskilde, Denmark) were coated for 1 h at 37°C with 100 μl of a solution of CK-B-derived peptides or rCK-B (10 μg/ml) in carbonate-bicarbonate buffer (15 mM Na_2_CO_3_, 35 mM NaHCO_3_, pH 9.6). After three washes with 100 mM phosphate buffer pH 7.4 (PT buffer; 5 min), the wells were incubated for 30 min with 200 μl of bovine serum albumin (BSA) diluted in PT buffer (5%, w/v; PT-BSA buffer) to saturate non-specific sites. After saturation, wells were incubated for 1 h with 100 μl of morphine, M6G, M3G or codeine, diluted in H_2_O at increasing concentrations. The plate was then washed three times with PT buffer, and 100 μl of the mouse primary anti-morphine antibody (which detects morphine, codeine, M3G and M6G according to the manufacturer’s antibody datasheet) diluted in PT-BSA buffer (1:2,000, v/v; 3A6, ref AMM00033; Aviva System Biology, San Diego, CA, USA) were added. After three more washes with PT buffer, 100 μl of the secondary antibody in PT-BSA buffer (HRP-conjugated donkey anti-mouse IgG, P.A.R.I.S. CliniSciences, Nanterre, France; 1:500, v/v) were added and incubated for 30 min at room temperature. After two washes with PT buffer, followed by two washes with a pH 7.5 phosphate-citrate-0.05% Tween 20 buffer (10 min), revelation was performed with 200 μl of a 1-Step Ultra TMB substrate solution (3,3′,5,5′ tetramethylbenzidine, Thermo Fischer Scientific). After 15 min of incubation at RT, the reaction was stopped by the addition of 50 μl of 2N hydrochloric acid. Optical density was determined at 450 nm with a Multiskan EX plate reader (Thermo Life Sciences, Cergy Pontoise, France). Each ELISA points was tested in triplicate in three independent experiments. All samples with a triplicate CV > 10% were retested to obtain a CV below or equal to 10%.

### Association Assays

Association assays were performed with 0.5 μg of the mouse rCK-B in the presence of 1 or 5 μg of morphine, in a final volume of 10 μl incubated during 15 min at 37°C. Samples were diluted 1:2 with the gel-loading buffer (see below).

### Gel Electrophoresis

Proteins were separated on SDS-PAGE gradient gels (4%–12% acrylamide; Novex, MES running buffer). Samples were suspended in 20 μl of loading buffer containing 60 mM Tris HCl pH 6.8, 2% SDS (w/v), 4 M urea, 5% glycerol (v/v), 5 mM EDTA, 1% β-mercaptoethanol (v/v) and 0.05% bromophenol blue (w/v) and subjected to heat treatment (100°C, 5 min). Gels were run in duplicate. One gel was silver-stained, and the other one was used for Western Blot analysis (see below).

### Silver Staining

Silver staining was done according to the manufacturer’s (Proteabio Europe, Langlade, France) instructions. Silver-stained gels and Western Blot analysis with an anti-CK antibody were used to assess the stability of CK after heat treatment. No difference in silver-stained band intensity or immunoreactivity was noticed in any experiment.

### Western Blot

After electrophoretic separation, the gel was electrotransferred onto a polyvinyldifluorene (PVDF) membrane (Bio-Rad). PVDF membranes were treated for 30 min with a saturation solution containing PBS, 5% BSA (w/v) and 0.05% Tween 20 (v/v). Then, PVDF membranes were incubated with the 3A6 mouse monoclonal anti-morphine primary antibody for 1 h at room temperature (1:1,000). The mouse monoclonal 3A6 anti-morphine antibodies (ref AMM00033; Aviva System Biology) were raised against a ((5 alpha 6 alpha) 7 8 didehydro-4 5 epoxy-17methylmorphinan-3 5diol)-BSA conjugate. Binding of the primary antibody was detected using HRP-conjugated donkey anti-mouse antiserum (P.A.R.I.S.; 1:50,000 in PBS, 5% of BSA (w/v), 0.05% Tween 20 (v/v)). Luminata FORTE™ (Millipore, France) was used as the substrate. Non-specific binding of the secondary antibody was ruled out by omitting the primary antibody: non-specific labeling was absent with the secondary antibody alone (data not shown).

### Peptide Synthesis

CK-B-derived peptides were synthesized by Proteogenix (Schiltigheim, France): CK-B_1–75_, CK-B_65–140_, CK-B_127–199_, CK-B_184–258_, CK-B_248–343_, CK-B_286–381_, CK-B_184–258_, CK-B_199–223_, CK-B_214–238_, CK-B_214–238_.

### Circular Dichroism

Circular dichroism spectra were recorded from 260 nm to 190 nm using a J-810 spectropolarimeter (Jasco, Tokyo, Japan). Spectral resolution and data pitch were adjusted to 1 nm while the scanning speed was set up at 50 nm/min. Samples containing 0.2 mg/mL of CK-B_1–75_ in H_2_O (pH 6) and CK-B_184–258_ (in 2.5 mM of NaOH, pH 7) were transferred into a quartz cuvette of 1 mm path length and maintained at the temperature of 23°C. The absence of aggregates was confirmed by a Dynamic Light Scattering analysis (DLS, Zetasizer NanoS; Malvern Panalytical, Malvern, UK). The secondary structures of the peptides were calculated from the spectra using a least squares fit procedure implemented in the DicroProt analysis software (Deléage and Geourjon, 1993).

### CK Activity Assay

We used a commercial creatine kinase activity assay kit (MAK116, Sigma-Aldrich) to measure the potential impact of morphine binding on CK-B enzymatic activity. The protocol followed the manufacturer’s instructions at room temperature. Briefly, 1 μg of rCK-B or 75 μg of brain extract were added per well (96-well plates, NUNC) with increasing concentrations of morphine (0, 0.01, 0.1, 1, 10, 25, 50, 100 μM final). The reconstituted reagent (made of assay buffer, enzyme mix and substrate solution) was then added to start the reaction. Fluorescence was recorded using a Mithras LB940 fluorescence plate reader (Berthold, Bad Wildbad, Germany), with excitation and emission filters of 350 nm and 460 nm, respectively. Four replicates were used for each data point.

### Statistics

Data were analyzed using Graphpad Prism Software 7.0. Non-parametric statistical tests were used due to low sample size and the non-Gaussian distribution of some groups. For *in vitro* rCK-B assays, data were expressed as percentage of the control (no morphine condition) to account for variability across replicates done on separate days. Statistical differences were tested with the Kruskal-Wallis test and Dunn’s multiple comparisons as *post hoc*. Brain extract assays were analyzed using the Mann-Whitney U test. Differences between groups were considered statistically significant at *p* < 0.05.

## Results

### Characterization of the CK-B Affinity for Morphine

**Figure 1:**
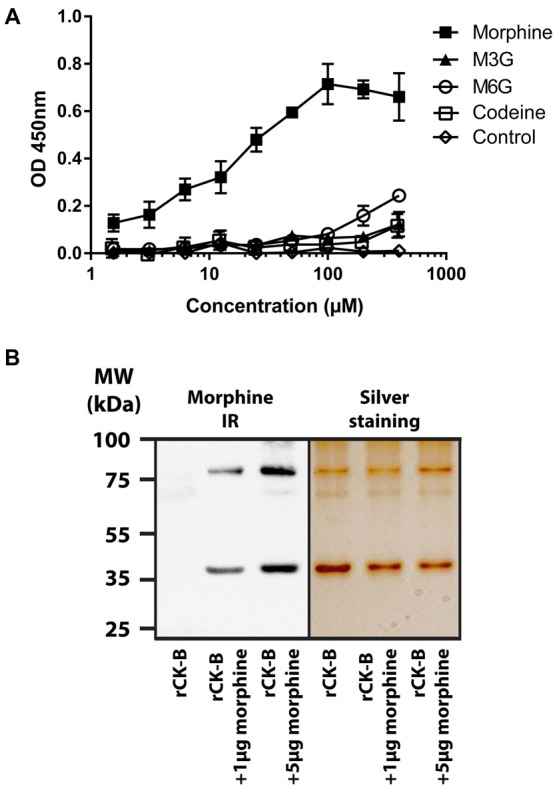
Characterization of the creatine kinase B (CK-B) affinity for morphine. **(A)** Affinity of mouse recombinant CK-B (rCK-B) for morphine, morphine-3-glucuronide (M3G) and morphine-6-glucuronide (M6G) using an immunoenzymatic assay (enzyme-linked immunosorbent assay, ELISA). ELISAs were performed with the 3A6 morphine antibody which detects morphine, codeine, M3G and M6G with the same affinity. Optical density increases with formation of peptide-alkaloid complexes. Data are expressed as Mean ± standard error of the mean (SEM) of triplicates (representative result of *n* = 3 independent experiments). **(B)** Characterization of CK-B-morphine complexes resistant to SDS-polyacrylamide gel electrophoresis (SDS-PAGE). Left panel, Western Blot analysis showing morphine-immunoreactivity (IR) after incubation of rCK-B with morphine (representative result of *n* = 3 independent experiments). Right panel, silver staining of a duplicate gel performed in parallel.

The binding of mouse rCK-B, produced in *E. coli* to increasing concentrations (1.56 μM to 400 μM) of morphine, codeine, M3G or M6G was tested by ELISA. The results show that mouse rCK-B binds morphine (Figure 1A) with a calculated Kd of 10.8 μM. Our results further revealed that M3G, M6G and codeine only weakly bind to rCK-B (Kd > 100 μM) compared to morphine. As a non-specific binding control, a series of wells were coated with BSA instead of rCK-B and incubated with increasing concentrations of morphine, M3G or M6G. No binding of morphine, M3G or M6G to BSA was observed (Figure 1A, control).

Together, these results indicate that rCK-B binds morphine with a micromolar range affinity.

### Characterization of a Morphine-rCK-B Complex Resistant to SDS-PAGE

Mouse rCK-B (0.5 μg) was incubated 15 min in the presence of 1 or 5 μg of morphine. Samples were resuspended in loading buffer containing both SDS and urea. A heating step (100°C; 5 min) was performed before polyacrylamide gel electrophoresis (PAGE).

Western Blot analysis using anti-morphine antibody showed that the mouse rCK-B alone is not labeled by the anti-morphine antibody (Figure 1B, left panel). Morphine immunolabeling was observed at 42 kDa (CK-B monomers) and 84 kDa (CK-B dimers) only when CK-B was incubated with 1 and 5 μg of morphine. As a control, an identical gel was run in parallel and silver-stained (Figure 1B, right panel). This control shows the presence of two bands at 42 kDa and 84 kDa in each condition. These two bands correspond to the monomers and dimers of mouse rCK-B, respectively.

These results indicate that morphine-mouse rCK-B complexes are resistant to SDS-PAGE.

### Identification of the CK-B Fragments Displaying an Affinity for Morphine

**Figure 2:**
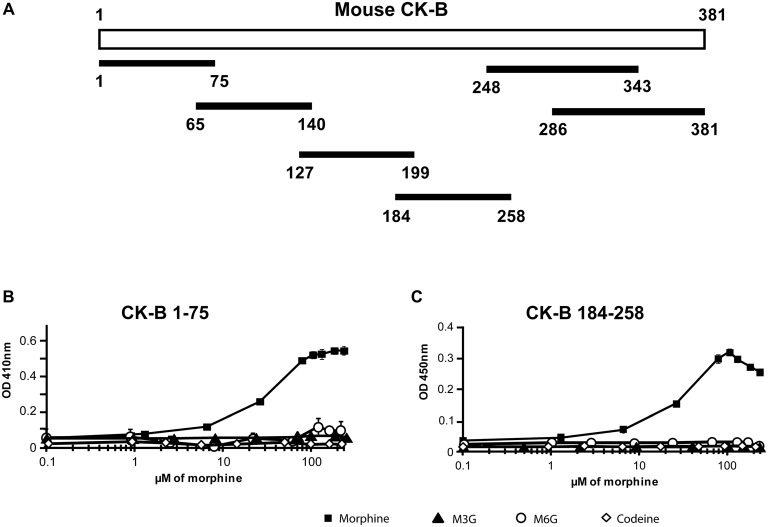
Affinity of synthetic peptides generated from mouse CK-B sequence for morphine and its derivatives. **(A)** Map of the six CK-B-derived peptides. **(B)** Immunoenzymatic (ELISA) determination of the binding properties of peptides CK-B_1–75_ and **(C)** CK-B_184–258_ to morphine, M6G, M3G and codeine. Data are expressed as Mean ± SEM of triplicates (representative result of *n* = 3 independent experiments). ELISAs were performed with the 3A6 anti-morphine antibody which binds morphine, codeine, M3G and M6G. Optical density increases with formation of peptide-alkaloid complexes.

To identify the morphine-binding motifs of CK, six overlapping peptides, namely CK-B_1–75_, CK-B_65–140_, CK-B_127–199_, CK-B_184–258_, CK-B_248–343_ and CK-B_286–381_ were tested for morphine, M3G, M6G and codeine binding *via* ELISA (Figure 2A). Increasing concentrations of opiates (0.1 μM to 324 μM) were added to wells pre-coated with the peptides (0.5 μg). CK-B_1–75_ and CK-B_184–258_ were able to bind morphine (Figures 2B,C) with a calculated Kd of 12.5 μM and 13.2 μM, respectively. However, ELISA analysis revealed that the six CK-derived peptides have no affinity for M3G, M6G or codeine (Figures 2B,C). Circular dichroism analysis revealed that in H_2_O, CK-B_1–75_ adopts a structure consisting of 3% alpha helix, 15% beta sheets and 82% random coils. Similarly, the calculated secondary structure of CK-B_184–258_ is 8% alpha helix, 19% beta sheets and 73% random coils. Focusing on the CK-B_184–258_ peptide, we tested the affinity of four smaller overlapping peptides (CK-B_184–258_, CK-B_199–223_, CK-B_214–238_, CK-B_214–238_). None of these four peptides displayed an affinity for morphine using an ELISA approach (data not shown).

Taken together, these results indicate that mouse CK-B_1–75_ and CK-B_184–258_ have a micromolar range affinity restricted to morphine, as observed for the mouse rCK-B.

### Characterization of the Effect of Morphine Binding on rCK-B Activity *in vitro*

**Figure 3:**
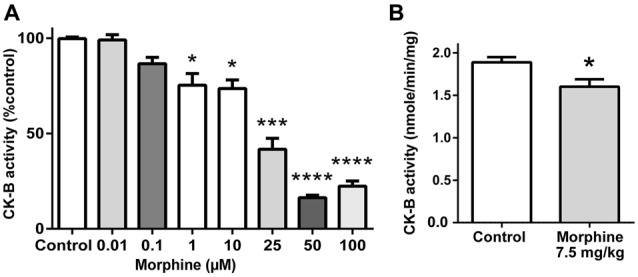
Effect of morphine on CK-B activity. **(A)** *In vitro* effect of increasing concentrations of morphine on CK-B activity. Data are expressed as Mean ± SEM. Kruskal-Wallis test with Dunn’s multiple comparison; *n* = 8; *p < 0.05; ***p < 0.001; ****p < 0.0001. **(B)** *In vivo* effect of an injection of saline or 7.5 mg/kg of morphine on brain CK-B activity (ATP production). Data are expressed as Mean ± SEM. Mann-Whitney test; *n* = 8 animals per group. *p < 0.05.

Next, we measured the potential impact of morphine binding to CK-B on the formation of ATP from phosphocreatine. Mouse rCK-B (1 μg) was incubated in the presence of increasing concentrations of morphine (0, 0.01, 0.1, 1, 10, 25, 50 and 100 μM; Figure 3A). The activity of rCK-B was monitored by measuring the production of ATP during 20 min. Morphine (1 μM) significantly decreased the formation of ATP by 27% (36.4 ± 1.8 nmol/min/mg) compared to the control condition (50 ±1.4 nmol/min/mg). Further increasing morphine concentrations dose-dependently inhibited CK-B activity until a maximal level of inhibition was reached (80% decrease in activity for morphine >50 μM). This experiment shows that 1 μM morphine, corresponding to a concentration close to the Kd of CK-B for morphine, significantly decreases CK-B activity *in vitro*.

### Characterization of the Effect of Morphine on CK-B Activity *in vivo*

In mice, the effect of morphine on CK-B activity *in vivo* was measured after a single injection of morphine (7.5 mg/kg, i.p.). Endogenous CK-B activity was tested on 75 μg of brain extract, by monitoring ATP production over 10 min. Results indicate that acute morphine treatment induces a statistically significant decrease of CK-B activity by 15.3% (1.60 ± 0.09 μmole/min/mg of total brain protein) compared to saline-treated animals (1.89 ± 0.06 μmole/min/mg of total brain protein; Figure 3B).

## Discussion

The present study reports that recombinant mouse rCK-B displays a micromolar affinity for morphine but not for M3G, M6G or codeine. Two morphine-binding sites were identified, namely peptides CK-B_1–75_ and CK-B_184–258_, which displayed a similar micromolar affinity for morphine. In addition, we show that CK-B-morphine complexes are resistant to detergents (SDS), chaotropic agents (urea), reducing agents and heat treatment. Finally, our results show that at a concentration that results in complex formation, morphine significantly decreases the activity of the mouse rCK-B *in vitro* and endogenous mouse brain CK-B *in vivo*.

### The Morphine-CK-B Complexes

CK-B is the main I2B site for imidazoline and derivatives (such as clonidine, guanfacine…; Gentili et al., 2006; Kimura et al., 2009; Li, 2017). In addition, CK was described to covalently bind dopamine (Van Laar et al., 2009), which is a morphine precursor in plants and animals (Laux-Biehlmann et al., 2013), and to form highly stable complexes with ASB9 (Kwon et al., 2010; Balasubramaniam et al., 2015). However, the potential binding of CK-B to morphine, as well as a potential modulatory role of morphine on CK-B activity, have never been studied.

Covalent binding of morphine to proteins has been first proposed by Misra and Mitchell (1971) and Misra et al. (1971). This group was able to demonstrate that a single subcutaneous injection of 10 mg/kg of [^14^C]-morphine-N-methyl led to morphine-related radioactivity lasting in the rat CNS for at least 3 weeks. Later, [C1-^3^H]-morphine and [N-^14^CH_3_]-morphine were found to irreversibly bind insoluble tissue components after administration (Mullis et al., 1979; Nagamatsu et al., 1983). In addition, S-glutathionylmorphine complexes have been characterized (Correia et al., 1984; Kumagai et al., 1990) and are likely formed first by the action of morphine-6-deshydrogenase (EC 1.1.1.218) leading to the formation of morphinone (Endo et al., 2013), followed by a spontaneous step allowing conjugation to glutathione (GSH) or proteins displaying cysteine residues. Besides, the binding of morphine to 35–50 kDa proteins in mice has also been reported (Nagamatsu et al., 1983; Nagamatsu and Hasegawa, 1992, 1993). Morphine binding to these unknown proteins was described as “covalent” because morphine-protein complexes were resistant to both methanol precipitation and SDS-PAGE denaturing conditions (Nagamatsu and Hasegawa, 1992).

In this article, we similarly unveil the formation of mouse rCK-B-morphine complexes that are resistant to SDS-PAGE treatment. Such a strong binding seems incompatible with the micromolar range affinity measured by ELISA. However, ELISA only reflects a biased affinity: (i) resulting from conditions that do not especially promote interactions of morphine with CK-B or its derived fragments; and (ii) dependent on the primary and secondary antibodies. This point is strengthened by the fact that both CK-B_1–75_ and CK-B_184–258_ display an affinity (μM range) similar to that of the full-length mouse rCK-B. In addition, the control condition performed with a nonspecific morphine-binding protein (i.e., BSA) did not result in any binding and therefore highlights the stringency of our ELISA (Judis, 1977; Leow et al., 1993). It is likely that morphine-binding sites are present in a well-defined structure of the peptides rather than in unstructured parts. Smaller CK-B_184–258_-derived peptides did not show any affinity for morphine, therefore we could not determine a specific morphine-binding site. This suggests that multiple structural parts all along the sequence of CK-B_184–258_ are required for the binding of morphine. The formation of strong morphine-CK-B complexes can be explained by two hypotheses: (i) non-covalent interactions involving ionic, hydrophobic and/or van der Waals interactions lead to a highly stable complex or (ii) a covalent binding of morphine to cysteine residues present in the CK-B occurs. However, our *in vitro* experimental conditions rule out a possible cross-linking involving cysteine residues (experiments performed in reducing conditions) and the action of morphine-6-deshydrogenase (reaction media do not contain this enzyme). Thus, it is likely that non-covalent complexes are formed with morphine and involve specific interactions as shown for the CK-ASB9 complexes (Balasubramaniam et al., 2015).

### CK-B Activity Modulation by Morphine

As CK-B represents a crucial enzyme that regulates ATP bioavailability in the brain, the ability of morphine to alter CK-B activity may dramatically affect CNS metabolism. Notably, the binding of the irreversible I2B ligand BU99006 (20 μM) to CK-B was shown to decrease the apparent Vmax of CK-B by up to 16% (Kimura et al., 2009). However, other I2B ligands (2-BFI, BU224, agmatine) did not affect CK-B activity. In addition, it was shown that a chronic administration of cannabidiol increases CK activity by up to 20% in the rat brain (Valvassori et al., 2013).

The implication of I2B sites in the modulation of morphine-induced analgesia has been well documented (Sanchez-Blázquez et al., 2000; Gentili et al., 2006; Ciubotariu and Nechifor, 2012; Caprioli et al., 2015) and the involvement of I2B sites in the modulation of morphine withdrawal and tolerance has been reported in rodents (Boronat et al., 1998; Ruiz-Durántez et al., 2003; Miralles et al., 2005). For example, the I2 ligand agmatine increases morphine-induced analgesia and inhibits tolerance and dependence to opioids (Dardonville et al., 2006).

In the brain, changes in the expression of energy metabolism enzymes contribute to behavioral abnormalities during chronic morphine treatment (Chen et al., 2007) but can also deeply impact gliotransmission through the modulation of ATP release (Harada et al., 2016). Accordingly, morphine-binding significantly decreases CK-B enzymatic activity by 27% when morphine is present at a concentration close to the Kd value. In addition, we have shown that *in vivo*, CK-B activity in brain tissue is decreased by 15 % after injection of an analgesic dose of morphine. Interestingly, our results are in agreement with the effect of morphine on ATP levels in the rat neocortex and thalamus, where respective decreases of 14% and 26% have been observed *in vivo* (Nasello et al., 1973).

Our results reveal two potential morphine-CK-B binding sites, namely CK-B_1–75_ and CK-B_184–258_. The former is not involved in CK-B activity. Conversely, CK-B_184–258_ contains a conserved, negatively charged amino acid cluster, Glu_226_, Glu_227_, Asp_228_ (EED) located in the active site of CK isoenzymes (Eder et al., 2000). This particular motif has been described as essential for CK enzymatic activity. Thus, we can hypothesize that morphine alters CK-B function through binding to the CK-B_184–258_ sequence. A limitation of our study is that we only evaluated a fixed time point after euthanizing the animals, and a different pattern of CK-B responses might occur at different time points. Further clarification of the impact of morphine on CK-B function *in vivo* is needed, e.g., through brain microdialysis and ATP quantification. Another open question is the impact of chronic morphine treatment in patients on CK-B activity, which is likely to differ from our acute morphine-exposure treatment. Indeed, a chronic morphine exposure might inhibit CK-B during a long period leading to important physiological changes and a measurable phenotype.

### *In vivo* Complex Formation

After administration, morphine rapidly reaches the CNS where it binds to mu opioid receptors (MORs) to produce analgesia. Then, morphine is taken up by glial cells including microglial cells and astrocytes through the action of the organic cation transporter OCT1 (Tzvetkov et al., 2013). After conversion by UDP-glucuronosyl-transferase (UGT) enzymes (King et al., 2000; Weinsanto et al., 2018), glucuronides are released through the efflux MRP3 transporter (multidrug resistance protein 3; Zelcer et al., 2005). In this context, it is plausible that after administration and cellular uptake, morphine can form complexes with cytoplasmic CK-B and therefore negatively modulate its activity. Interestingly, injection (i.v., i.p. or s.c.) of an analgesic dose of morphine (10 to 20 mg/kg) to mice results in serum concentrations ranging from 2 μM to 35 μM. Similarly, morphine content in the brain reaches nmol/g of tissue levels (corresponding to a μM range), indicating that our findings are relevant *in vivo* (Patrick et al., 1975; Vekovischeva et al., 2001; Handal et al., 2002; Xie et al., 2017).

Endogenous morphine has been detected in numerous cerebral areas and brain cells of different species including the mouse (Laux et al., 2011, 2012; Laux-Biehlmann et al., 2013). Specifically, endogenous morphine is detected in GABA-basket cells, astrocytes and microglial cells (Muller et al., 2008; Laux et al., 2011, 2012; Togna et al., 2013). In addition, an increase of endogenous morphine and M6G blood levels has been linked to stress (Meijerink et al., 1999; Brix-Christensen et al., 2000; Goumon et al., 2000) and pathologies such as sepsis, with concentrations reaching up to 0.25 μM (Glattard et al., 2010; Laux-Biehlmann et al., 2012). In contrast to opioidergic peptides (e.g., enkephalins), the functional role of endogenous morphine in the CNS remains to be elucidated. Endogenous morphine is found in the cytoplasm of astrocytes expressing both OCT1 (Schreiber et al., 1990) and CK-B (Tachikawa et al., 2004). Although endogenous morphine concentrations are unlikely to reach 1 μM, uptake by astrocytic processes could theoretically bring endogenous morphine in contact with cytoplasmic CK-B. Whether endogenous morphine-CK-B complexes exist and might influence bioavailability of ATP in astrocytes and impact ATP-mediated gliotransmission through CK-B inhibition remains to be investigated in further studies.

### Clinical Relevance of Morphine-CK-B Interactions

The *in vitro* and *in vivo* impact of morphine on CK-B activity in the mouse raises the question of a potential relevance of morphine-CK-B interactions in the case of clinical or recreational use of morphine or its derivatives in humans.

First, doses administered to morphine-naive humans are usually two orders of magnitude lower than those needed to elicit analgesia in rodents. Thus, peak morphine blood concentrations (and presumably tissue concentrations) after oral therapeutic dosing typically only reach 100 nM (Klimas and Mikus, 2014), a concentration far below the 1 μM of morphine needed for CK-B modulation.

However, it is possible that intracellular morphine could reach micromolar concentrations in chronically treated, tolerant patients. Indeed, dose escalation following long-term treatment can lead to patients receiving 500 mg of morphine or more per day. Thus, in the case of chronic use of morphine or codeine, whether in clinical (e.g., cancer patients) or drug abuse settings, morphine concentrations rise to low μM levels in both plasma and brain (Säwe et al., 1983; Frost et al., 2016). In addition, morphine concentrations in the cerebrospinal fluid (CSF) and brain extracellular fluid (ECF) have been reported to reach nM to μM values in different conditions, including epidural injection during chronic intractable pain due to cancer and codeine overdoses (Coombs et al., 1985; Bouw et al., 2001; Ederoth et al., 2004).

It is also important to mention the fact that M3G concentrations in these cases are far higher than those of morphine. CK-B is an intracellular protein highly expressed in astrocytes which are known to metabolize morphine into M3G (Suleman et al., 1998; Heurtaux et al., 2006; Sabolovic et al., 2007; Ouzzine et al., 2014). The active uptake of morphine by astrocytes might allow intracellular morphine to reach μM levels. Unfortunately, intracellular morphine concentrations have never been measured in the human brain, although it has been shown in rodents that morphine preferentially accumulates inside cells, especially astrocytes rather than in the ECF or CSF (Stain-Texier et al., 1999; Ouzzine et al., 2014).

In conclusion, CK-B/morphine interactions have the potential to be of clinical relevance and this warrants further investigation. While morphine concentrations may not reach sufficient levels to significantly impact CK-B activity in normal dosing regimens, chronic administration and high doses administered to tolerant patients might affect ATP bioavailability in humans.

## Conclusion

Taken together, our results unveil a strong interaction between CK-B and morphine, occurring *in vitro* and *in vivo*. This interaction negatively impacts CK-B activity and thus has the potential to affect energy metabolism and glial transmission. Whether or not morphine is able to bind to and modulate CK-M or mitochondrial CK remains to be studied, but this seems likely given a high sequence homology between these enzymes and CK-B. Additional experiments will allow to further elucidate the binding site of morphine and study, in detail, the consequences of this interaction. The impact of CK-B and its derived peptides on morphine analgesia and metabolism will be investigated in the future. Finally, a detailed study has to focus on endogenous morphine in order to determine its potential implication in energy bioavailability in both physiological and pathological processes.

## Author Contributions

YG, IW, AL-B, JM, M-OP, SC and AC: conceptualization. IW, AL-B, JM, VC, FD, FG, AM and YG: methodology. IW, AL-B, JM, VC, FD, AM and YG: investigation. YG, IW, FG, SC, M-OP, AM and AC: writing, review and editing. YG: funding acquisition, resources and supervision.

## Conflict of Interest Statement

The authors declare that the research was conducted in the absence of any commercial or financial relationships that could be construed as a potential conflict of interest.

## Abbreviations

ASB9: ankyrin repeat and SOCS box protein 9
CK-B: brain creatine kinase
CK-M: muscular creatine kinase
CNS: central nervous system
I2B: I2-binding
M3G: morphine-3-glucuronide
M6G: morphine-6-glucuronide
PEBP: phosphatidylethanolamine-binding protein
SEM: standard error of the mean.

## References

[B1] Andersen G. Christrup L. Sjogren P. (2003). Relationships among morphine metabolism, pain and side effects during long-term treatment: an update. J. Pain Symptom. Manage. 25, 74–91. 10.1016/s0885-3924(02)00531-6 12565191

[B2] Atmanene C. Laux A. Glattard E. Muller A. Schoentgen F. Metz-Boutigue M.-H.. (2009). Characterization of human and bovine phosphatidylethanolamine-binding protein (PEBP/RKIP) interactions with morphine and morphine-glucuronides determined by noncovalent mass spectrometry. Med. Sci. Monit. 15, BR178–BR87. 19564817

[B3] Balasubramaniam D. Schiffer J. Parnell J. Mir S. P. Amaro R. E. Komives E. A. (2015). How the ankyrin and SOCS box protein, ASB9, binds to creatine kinase. Biochemistry 54, 1673–1680. 10.1021/bi501420n 25654263 PMC4348336

[B4] Boronat M. A. Olmos G. García-Sevilla J. A. (1998). Attenuation of tolerance to opioid-induced antinociception and protection against morphine-induced decrease of neurofilament proteins by idazoxan and other I2-imidazoline ligands. Br. J. Pharmacol. 125, 175–185. 10.1038/sj.bjp.0702031 9776358 PMC1565592

[B5] Bouw R. Ederoth P. Lundberg J. Ungerstedt U. Nordström C. H. Hammarlund-Udenaes M. (2001). Increased blood-brain barrier permeability of morphine in a patient with severe brain lesions as determined by microdialysis. Acta Anaesthesiol. Scand. 45, 390–392. 10.1034/j.1399-6576.2001.045003390.x 11207479

[B6] Brix-Christensen V. Goumon Y. Tønnesen E. Chew M. Bilfinger T. Stefano G. B. (2000). Endogenous morphine is produced in response to cardiopulmonary bypass in neonatal pigs. Acta Anaesthesiol. Scand. 44, 1204–1208. 10.1034/j.1399-6576.2000.441004.x 11065199

[B7] Caprioli G. Mammoli V. Ricciutelli M. Sagratini G. Ubaldi M. Domi E.. (2015). Biological profile and bioavailability of imidazoline compounds on morphine tolerance modulation. Eur. J. Pharmacol. 769, 219–224. 10.1016/j.ejphar.2015.11.021 26593429

[B8] Chen X.-L. Lu G. Gong Y.-X. Zhao L.-C. Chen J. Chi Z.-Q.. (2007). Expression changes of hippocampal energy metabolism enzymes contribute to behavioural abnormalities during chronic morphine treatment. Cell Res. 17, 689–700. 10.1038/cr.2007.63 17667915

[B9] Ciubotariu D. Nechifor M. (2012). Involvement of imidazoline system in drug addiction. Rev. Med. Chir. Soc. Med. Nat. Iasi 116, 1118–1122. 23700899

[B10] Coombs D. W. Fratkin J. D. Meier F. A. Nierenberg D. W. Saunders R. L. (1985). Neuropathologic lesions and CSF morphine concentrations during chronic continuous intraspinal morphine infusion. A clinical and post-mortem study. Pain 22, 337–351. 10.1016/0304-3959(85)90040-5 2413419

[B11] Correia M. A. Krowech G. Caldera-Munoz P. Yee S. L. Straub K. Castagnoli N. Jr. (1984). Morphine metabolism revisited. II. Isolation and chemical characterization of a glutathionylmorphine adduct from rat liver microsomal preparations. Chem. Biol. Interact. 51, 13–24. 10.1016/0009-2797(84)90016-4 6547643

[B12] Dardonville C. Fernandez-Fernandez C. Gibbons S. L. Ryan G. J. Jagerovic N. Gabilondo A. M.. (2006). Synthesis and pharmacological studies of new hybrid derivatives of fentanyl active at the μ-opioid receptor and I_2_-imidazoline binding sites. Bioorg. Med. Chem. 14, 6570–6580. 10.1016/j.bmc.2006.06.007 16797997

[B13] Deléage G. Geourjon C. (1993). An interactive graphic program for calculating the secondary structure content of proteins from circular dichroism spectrum. Comput. Appl. Biosci. 9, 197–199. 10.1093/bioinformatics/9.2.197 8481823

[B14] Eder M. Stolz M. Wallimann T. Schlattner U. (2000). A conserved negatively charged cluster in the active site of creatine kinase is critical for enzymatic activity. J. Biol. Chem. 275, 27094–27099. 10.1074/jbc.M004071200 10829032

[B15] Ederoth P. Tunblad K. Bouw R. Lundberg C. J. Ungerstedt U. Nordström C. H.. (2004). Blood-brain barrier transport of morphine in patients with severe brain trauma. Br. J. Clin. Pharmacol. 57, 427–435. 10.1046/j.1365-2125.2003.02032.x 15025740 PMC1884477

[B16] Endo S. Matsunaga T. Fujimoto A. Kumada S. Arai Y. Miura Y.. (2013). Characterization of rabbit morphine 6-dehydrogenase and two NAD^+^-dependent 3α(17β)-hydroxysteroid dehydrogenases. Arch. Biochem. Biophys. 529, 131–139. 10.1016/j.abb.2012.11.013 23228597

[B17] Frost J. Løkken T. N. Helland A. Nordrum I. S. Slørdal L. (2016). Post-mortem levels and tissue distribution of codeine, codeine-6-glucuronide, norcodeine, morphine and morphine glucuronides in a series of codeine-related deaths. Forensic Sci. Int. 262, 128–137. 10.1016/j.forsciint.2016.02.051 26986973

[B18] Gentili F. Cardinaletti C. Carrieri A. Ghelfi F. Mattioli L. Perfumi M.. (2006). Involvement of I2-imidazoline binding sites in positive and negative morphine analgesia modulatory effects. Eur. J. Pharmacol. 553, 73–81. 10.1016/j.ejphar.2006.09.031 17081513

[B19] Glattard E. Welters I. D. Lavaux T. Muller A. H. Laux A. Zhang D.. (2010). Endogenous morphine levels are increased in sepsis: a partial implication of neutrophils. PLoS One 5:e8791. 10.1371/journal.pone.0008791 20098709 PMC2808358

[B20] Goumon Y. Angelone T. Schoentgen F. Chasserot-Golaz S. Almas B. Fukami M. M.. (2004). The hippocampal cholinergic neurostimulating peptide, the N-terminal fragment of the secreted phosphatidylethanolamine-binding protein, possesses a new biological activity on cardiac physiology. J. Biol. Chem. 279, 13054–13064. 10.1074/jbc.m308533200 14724289

[B21] Goumon Y. Bouret S. Casares F. Zhu W. Beauvillain J. C. Stefano G. B. (2000). Lipopolysaccharide increases endogenous morphine levels in rat brain. Neurosci. Lett. 293, 135–138. 10.1016/s0304-3940(00)01507-x 11027852

[B22] Handal M. Grung M. Skurtveit S. Ripel A. Mørland J. (2002). Pharmacokinetic differences of morphine and morphine-glucuronides are reflected in locomotor activity. Pharmacol. Biochem. Behav. 73, 883–892. 10.1016/s0091-3057(02)00925-5 12213535

[B23] Harada K. Kamiya T. Tsuboi T. (2016). Gliotransmitter release from astrocytes: functional, developmental, and pathological implications in the brain. Front. Neurosci. 9:499. 10.3389/fnins.2015.00499 26793048 PMC4709856

[B24] Heurtaux T. Benani A. Moulin D. Muller N. Netter P. Minn A. (2006). Induction of UGT1A6 isoform by inflammatory conditions in rat astrocytes. Neuropharmacology 50, 317–328. 10.1016/j.neuropharm.2005.09.007 16274708

[B25] Judis J. (1977). Binding of codeine, morphine, and methadone to human serum proteins. J. Pharm. Sci. 66, 802–806. 10.1002/jps.2600660615 874779

[B26] Kimura A. Tyacke R. J. Robinson J. J. Husbands S. M. Minchin M. C. Nutt D. J.. (2009). Identification of an imidazoline binding protein: creatine kinase and an imidazoline-2 binding site. Brain Res. 1279, 21–28. 10.1016/j.brainres.2009.04.044 19410564 PMC2722693

[B27] King C. D. Rios G. R. Green M. D. Tephly T. R. (2000). UDP-glucuronosyltransferases. Curr. Drug Metab. 1, 143–161. 10.2174/1389200003339171 11465080

[B28] Klimas R. Mikus G. (2014). Morphine-6-glucuronide is responsible for the analgesic effect after morphine administration: a quantitative review of morphine, morphine-6-glucuronide and morphine-3-glucuronide. Br. J. Anaesth. 113, 935–944. 10.1093/bja/aeu186 24985077

[B29] Kumagai Y. Todaka T. Toki S. (1990). A new metabolic pathway of morphine: *in vivo* and *in vitro* formation of morphinone and morphine-glutathione adduct in guinea pig. J. Pharmacol. Exp. Ther. 255, 504–510. 1700815

[B30] Kwon S. Kim D. Rhee J. W. Park J. A. Kim D. W. Kim D. S.. (2010). ASB9 interacts with ubiquitous mitochondrial creatine kinase and inhibits mitochondrial function. BMC Biol. 8:23. 10.1186/1741-7007-8-23 20302626 PMC2852384

[B31] Laux A. Delalande F. Mouheiche J. Stuber D. Van Dorsselaer A. Bianchi E.. (2012). Localization of endogenous morphine-like compounds in the mouse spinal cord. J. Comp. Neurol. 520, 1547–1561. 10.1002/cne.22811 22102217

[B32] Laux A. Muller A. H. Miehe M. Dirrig-Grosch S. Deloulme J. C. Delalande F.. (2011). Mapping of endogenous morphine-like compounds in the adult mouse brain: evidence of their localization in astrocytes and GABAergic cells. J. Comp. Neurol. 519, 2390–2416. 10.1002/cne.22633 21456021

[B33] Laux-Biehlmann A. Grafe N. Mouheiche J. Stuber D. Welters I. D. Delalande F.. (2012). Comparison of serum and lithium-heparinate plasma for the accurate measurements of endogenous and exogenous morphine concentrations. Br. J. Clin. Pharmacol. 74, 381–383. 10.1111/j.1365-2125.2012.04199.x 22295933 PMC3630759

[B34] Laux-Biehlmann A. Mouheiche J. Vérièpe J. Goumon Y. (2013). Endogenous morphine and its metabolites in mammals: history, synthesis, localization and perspectives. Neuroscience 233, 95–117. 10.1016/j.neuroscience.2012.12.013 23266549

[B35] Leow K. P. Wright A. W. Cramond T. Smith M. T. (1993). Determination of the serum protein binding of oxycodone and morphine using ultrafiltration. Ther. Drug Monit. 15, 440–447. 10.1097/00007691-199310000-00014 8249052

[B36] Li J. X. (2017). Imidazoline I2 receptors: an update. Pharmacol. Ther. 178, 48–56. 10.1016/j.pharmthera.2017.03.009 28322973 PMC5600648

[B37] Manos P. Bryan G. K. (1993). Cellular and subcellular compartmentation of creatine-kinase in brain. Dev. Neurosci. 15, 271–279. 10.1159/000111344 7805579

[B38] McGrath J. C. Drummond G. B. McLachlan E. M. Kilkenny C. Wainwright C. L. (2010). Guidelines for reporting experiments involving animals: the ARRIVE guidelines. Br. J. Pharmacol. 160, 1573–1576. 10.1111/j.1476-5381.2010.00873.x 20649560 PMC2936829

[B39] Meijerink W. J. Molina P. E. Abumrad N. N. (1999). Mammalian opiate alkaloid synthesis: lessons derived from plant biochemistry. Shock 12, 165–173. 10.1097/00024382-199909000-00001 10485593

[B40] Miralles A. Esteban S. Sastre-Coll A. Moranta D. Asensio V. J. Garcia-Sevilla J. A. (2005). High-affinity binding of β-carbolines to imidazoline I_2B_ receptors and MAO-A in rat tissues: norharman blocks the effect of morphine withdrawal on DOPA/noradrenaline synthesis in the brain. Eur. J. Pharmacol. 518, 234–242. 10.1016/j.ejphar.2005.06.023 16061219

[B41] Misra A. L. Mitchell C. L. (1971). Metal ion-catalysed interaction of peroxidase with morphine and protein. Experientia 27, 1442–1444. 10.1007/bf02154278 5144856

[B42] Misra A. L. Mitchell C. L. Woods L. A. (1971). Persistence of morphine in central nervous system of rats after a singel injection and its bearing on tolerance. Nature 232, 48–50. 10.1038/232048a0 4933173

[B43] Muller A. Glattard E. Taleb O. Kemmel V. Laux A. Miehe M.. (2008). Endogenous morphine in SH-SY5Y cells and the mouse cerebellum. PLoS One 3:e1641. 10.1371/journal.pone.0001641 18327293 PMC2265639

[B44] Mullis K. B. Perry D. C. Finn A. M. Stafford B. Sadée W. (1979). Morphine persistence in rat brain and serum after single doses. J. Pharmacol. Exp. Ther. 208, 228–231. 762653

[B45] Nagamatsu K. Hasegawa A. (1992). Covalent binding of morphine to isolated rat hepatocytes. Biochem. Pharmacol. 43, 2631–2635. 10.1016/0006-2952(92)90152-9 1632819

[B46] Nagamatsu K. Hasegawa A. (1993). Effect of sodium selenite on morphine-induced hepatotoxicity in mice. Drug Chem. Toxicol. 16, 241–253. 10.3109/01480549309081818 8404545

[B47] Nagamatsu K. Kido Y. Terao T. Ishida T. Toki S. (1983). Studies on the mechanism of covalent binding of morphine metabolites to proteins in mouse. Drug Metab. Dispos. 11, 190–194. 6135574

[B48] Nasello A. G. Depiante R. Tannhauser M. Izquierdo I. (1973). Effect of morphine on the RNA and ATP concentration of brain structures of the rat. Pharmacology 10, 56–59. 10.1159/000136422 4751542

[B49] Ouzzine M. Gulberti S. Ramalanjaona N. Magdalou J. Fournel-Gigleux S. (2014). The UDP-glucuronosyltransferases of the blood-brain barrier: their role in drug metabolism and detoxication. Front. Cell. Neurosci. 8:349. 10.3389/fncel.2014.00349 25389387 PMC4211562

[B50] Patrick G. A. Dewey W. L. Spaulding T. C. Harris L. S. (1975). Relationship of brain morphine levels to analgesic activity in acutely treated mice and rats and in pellet implanted mice. J. Pharmacol. Exp. Ther. 193, 876–883. 1151736

[B51] Ruiz-Durántez E. Torrecilla M. Pineda J. Ugedo L. (2003). Attenuation of acute and chronic effects of morphine by the imidazoline receptor ligand 2–(2-benzofuranyl)-2-imidazoline in rat locus coeruleus neurons. Br. J. Pharmacol. 138, 494–500. 10.1038/sj.bjp.0705052 12569074 PMC1573679

[B52] Sabolovic N. Heurtaux T. Humbert A. C. Krisa S. Magdalou J. (2007). *cis*- and *trans*-Resveratrol are glucuronidated in rat brain, olfactory mucosa and cultured astrocytes. Pharmacology 80, 185–192. 10.1159/000104149 17579296

[B53] Sanchez-Blázquez P. Boronat M. A. Olmos G. García-Sevilla J. A. Garzón J. (2000). Activation of I_2_-imidazoline receptors enhances supraspinal morphine analgesia in mice: a model to detect agonist and antagonist activities at these receptors. Br. J. Pharmacol. 130, 146–152. 10.1038/sj.bjp.0703294 10781010 PMC1572044

[B54] Säwe J. Svensson J. O. Rane A. (1983). Morphine metabolism in cancer patients on increasing oral doses—no evidence for autoinduction or dose-dependence. Br. J. Clin. Pharmacol. 16, 85–93. 10.1111/j.1365-2125.1983.tb02148.x 6882627 PMC1427961

[B55] Schreiber E. Harshman K. Kemler I. Malipiero U. Schaffner W. Fontana A. (1990). Astrocytes and glioblastoma cells express novel octamer-DNA binding proteins distinct from the ubiquitous Oct-1 and B cell type Oct-2 proteins. Nucleic Acids Res. 18, 5495–5503. 10.1093/nar/18.18.5495 2216722 PMC332229

[B56] Stain-Texier F. Boschi G. Sandouk P. Scherrmann J. M. (1999). Elevated concentrations of morphine 6-β-D-glucuronide in brain extracellular fluid despite low blood-brain barrier permeability. Br. J. Pharmacol. 128, 917–924. 10.1038/sj.bjp.0702873 10556926 PMC1571713

[B57] Suleman F. G. Abid A. Gradinaru D. Daval J. L. Magdalou J. Minn A. (1998). Identification of the uridine diphosphate glucuronosyltransferase isoform UGT1A6 in rat brain and in primary cultures of neurons and astrocytes. Arch. Biochem. Biophys. 358, 63–67. 10.1006/abbi.1998.0842 9750165

[B58] Tachikawa M. Fukaya M. Terasaki T. Ohtsuki S. Watanabe M. (2004). Distinct cellular expressions of creatine synthetic enzyme GAMT and creatine kinases uCK-Mi and CK-B suggest a novel neuron-glial relationship for brain energy homeostasis. Eur. J. Neurosci. 20, 144–160. 10.1111/j.1460-9568.2004.03478.x 15245487

[B59] Togna A. R. Antonilli L. Dovizio M. Salemme A. De Carolis L. Togna G. I.. (2013). *In vitro* morphine metabolism by rat microglia. Neuropharmacology 75C, 391–398. 10.1016/j.neuropharm.2013.08.019 23988259

[B60] Tzvetkov M. V. dos Santos Pereira J. N. Meineke I. Saadatmand A. R. Stingl J. C. Brockmoeller J. (2013). Morphine is a substrate of the organic cation transporter OCT1 and polymorphisms in *OCT1* gene affect morphine pharmacokinetics after codeine administration. Biochem. Pharmacol. 86, 666–678. 10.1016/j.bcp.2013.06.019 23835420

[B61] Valvassori S. S. Bavaresco D. V. Scaini G. Varela R. B. Streck E. L. Chagas M. H.. (2013). Acute and chronic administration of cannabidiol increases mitochondrial complex and creatine kinase activity in the rat brain. Braz. J. Psychiatry 35, 380–386. 10.1590/1516-4446-2012-0886 24402213

[B62] Van Laar V. S. Mishizen A. J. Cascio M. Hastings T. G. (2009). Proteomic identification of dopamine-conjugated proteins from isolated rat brain mitochondria and SH-SY5Y cells. Neurobiol. Dis. 34, 487–500. 10.1016/j.nbd.2009.03.004 19332121 PMC2759724

[B63] Vekovischeva O. Y. Zamanillo D. Echenko O. Seppälä T. Uusi-Oukari M. Honkanen A.. (2001). Morphine-induced dependence and sensitization are altered in mice deficient in AMPA-type glutamate receptor-A subunits. J. Neurosci. 21, 4451–4459. 10.1523/JNEUROSCI.21-12-04451.2001 11404432 PMC6762742

[B64] Weinsanto I. Laux-Biehlmann A. Mouheiche J. Maduna T. Delalande F. Chavant V.. (2018). Stable isotope-labelled morphine to study *in vivo* central and peripheral morphine glucuronidation and brain transport in tolerant mice. Br. J. Pharmacol. 175, 3844–3856. 10.1111/bph.14454 30051501 PMC6135784

[B65] Wyss M. Kaddurah-Daouk R. (2000). Creatine and creatinine metabolism. Physiol. Rev. 80, 1107–1213. 10.1152/physrev.2000.80.3.1107 10893433

[B66] Xie N. Gomes F. P. Deora V. Gregory K. Vithanage T. Nassar Z. D.. (2017). Activation of mu-opioid receptor and Toll-like receptor 4 by plasma from morphine-treated mice. Brain Behav. Immun. 61, 244–258. 10.1016/j.bbi.2016.12.002 27939249

[B67] Zelcer N. van de Wetering K. Hillebrand M. Sarton E. Kuil A. Wielinga P. R.. (2005). Mice lacking multidrug resistance protein 3 show altered morphine pharmacokinetics and morphine-6-glucuronide antinociception. Proc. Natl. Acad. Sci. U S A 102, 7274–7279. 10.1073/pnas.0502530102 15886284 PMC1091780

