# Corrigendum: Morphine Binds Creatine Kinase B and Inhibits Its Activity

**DOI:** 10.3389/fncel.2019.00292

**Published:** 2019-07-02

**Authors:** Ivan Weinsanto, Jinane Mouheiche, Alexis Laux-Biehlmann, François Delalande, Arnaud Marquette, Virginie Chavant, Florian Gabel, Sarah Cianferani, Alexandre Charlet, Marie-Odile Parat, Yannick Goumon

**Affiliations:** ^1^Institut des Neurosciences Cellulaires et Intégratives, CNRS UPR3212 and Université de Strasbourg, Strasbourg, France; ^2^Laboratoire de Spectrométrie de Masse BioOrganique, IPHC-DSA, CNRS UMR7178 and Université de Strasbourg, Strasbourg, France; ^3^CNRS UMR7177 and Université de Strasbourg, Strasbourg, France; ^4^Mass Spectrometry Facilities of the CNRS UPR3212, Strasbourg, France; ^5^School of Pharmacy, University of Queensland, PACE, Woolloongabba, QLD, Australia

**Keywords:** morphine, complex, ligand-binding protein, creatine kinase, high affinity

In the published article, there was an error in affiliation “2.” Instead of “Global Drug Discovery, Global Therapeutic Research Groups, Gynecological Therapies, Bayer Healthcare, Berlin, Germany”, it should be “Laboratoire de Spectrométrie de Masse BioOrganique, IPHC-DSA, CNRS UMR7178 and Université de Strasbourg, Strasbourg, France.”

Additionally, the current address of one author has been added as a footnote and is denoted using “‡.”

The authors apologize for this error and state that this does not change the scientific conclusions of the article in any way. The original article has been updated.

